# Tibialis anterior rerouting combined with calcaneal lengthening osteotomy as a single-stage reconstruction of symptomatic flexible flatfoot in children and adolescents

**DOI:** 10.1186/s13018-023-03890-7

**Published:** 2023-06-13

**Authors:** Samy Abdel-Hady Sakr, Ahmed Ibrahim Zayda, Mohamed Kamal Mesregah, Ahmed Abdelazim Abosalem

**Affiliations:** Department of Orthopaedic Surgery, Faculty of Medicine, Menoufia University, Shebin-El-Kom, Menoufia Egypt

**Keywords:** Pes planovalgus, Flexible flatfoot, Children, Calcaneal lengthening, Tibialis anterior rerouting, Gastrocnemius contracture

## Abstract

**Background:**

Symptomatic flexible flatfoot in children and adolescents should be surgically managed only if conservative measures have failed. The aim of this study was to assess functional and radiological results of tibialis anterior rerouting combined with calcaneal lengthening osteotomy as s single-stage reconstruction of symptomatic flexible flatfoot.

**Methods:**

The current study was a prospective study of patients with symptomatic flexible flatfoot treated by single-stage reconstruction in the form of tibialis anterior tendon rerouting combined with calcaneal lengthening osteotomy. The American Orthopaedic Foot and Ankle Society score (AOFAS) was utilized to evaluate the functional outcomes. The evaluated radiological parameters included the standing anteroposterior (AP) and lateral talo-first metatarsal angle, talar head coverage angle, and calcaneal pitch angle.

**Results:**

The current study included 16 patients (28 feet) with a mean age of 11.6 ± 2.1 years. There was a statistically significant improvement in the mean AOFAS score from 51.6 ± 5.5 preoperatively to 85.3 ± 10.2 at final follow-up. Postoperatively, there was a statistically significant reduction in the mean AP talar head coverage angle from 13.6 ± 4.4° to 3.9 ± 3°, the mean AP talo-first metatarsal angle from 16.9 ± 4.4° to 4.5 ± 3.6°, and the mean lateral talo-first metatarsal angle from 19.2 ± 4.9° to 4.6 ± 3.2°, *P* < 0.001. Additionally, the mean calcaneal pitch angle increased significantly from 9.6 ± 1.9° to 23.8 ± 4.8°, *P* < 0.001. Superficial wound infection occurred in three feet and was treated adequately by dressing and antibiotics.

**Conclusion:**

Symptomatic flexible flatfoot in children and adolescents can be treated with combined lateral column lengthening and tibialis anterior rerouting with satisfactory radiological and clinical outcomes.

*Level of evidence* Level IV.

## Background

Flatfoot deformity involves multiple components of medial arch collapse, hindfoot valgus, and forefoot abduction [1, 2]. Pediatric flatfoot can be rigid or flexible. Flexible flatfoot may be symptomatic or non-symptomatic, with preserved medial arch with non-weight bearing and arch flattening during stance [3, 4].

Asymptomatic patients follow a natural course of improvement over time and require periodic monitoring for signs of progression. In contrast, symptomatic forms can present with foot, leg, or knee pain, tight Achilles tendon, and walking difficulties [5–7].

Initially, activity modifications and orthotics are prescribed. In addition, nonsteroidal anti-inflammatory drugs (NSAIDs) can be used in more severe cases [8, 9]. Surgical intervention can be considered if the clinical response is unsatisfactory and all nonsurgical treatment options have been exhausted. Several surgical techniques have been described to correct these deformities, including soft tissue and bony procedures, with varying results [10–13].

As reported by Evans [14] and modified by Mosca [15], lateral column lengthening remains the most reliable option in children as it does not involve fusion, and it corrects both hindfoot and midfoot deformities [16, 17].

However, Evan’s procedure does not address the problems of the medial column, including forefoot supination. Therefore, Evan’s osteotomy is commonly combined with medial column procedures such as Young tenosuspension, tibialis posterior repositioning, tibialis anterior tendon transfer, or talonavicular joint capsular plication [10, 18–20].

One of the procedures that address the medial arch problems is tibialis anterior tendon plantar rerouting to serve as a plantar muscle and as a support to the talar head [21].

The current study aimed to assess the clinical and radiological results of single-stage reconstruction of symptomatic flexible flatfoot in pediatrics and adolescents using lateral column lengthening osteotomy and tibialis anterior tendon plantar rerouting.

## Methods

This study was a prospective case series of children and adolescents with symptomatic flexible flatfoot treated by single-stage reconstruction using rerouting of the tibialis anterior tendon combined with calcaneal lengthening osteotomy from January 2014 to April 2016. A single pediatric orthopedic surgeon operated on all patients at the Pediatric Unit of the Orthopedic Surgery Department, Menoufia University Faculty of Medicine, after approval of the Institutional Review Board (IRB) and written consent of all patients’ parents.

Inclusion criteria were children and adolescents between 9 to 15 years with idiopathic symptomatic flexible flatfoot that failed to respond to conservative management for at least 3 months, no previous reconstructive surgery for the affected foot, and no local or general contraindications for surgery. Patients had to complete at least 2 years of follow-up to be included in this investigation. Rigid flatfoot and patients with associated neuromuscular disorders or syndromes were excluded.

### Preoperative evaluation

Patients were evaluated preoperatively, both generally, locally, and radiologically. Generally, to assess body build, fitness for surgery, and associated neuromuscular disorders or syndromes. Locally, to evaluate the type of flatfoot, whether flexible or rigid using the Hubscher maneuver (Jack’s test) and the soft tissue condition of the foot.

Radiologically, standing anteroposterior (AP) and lateral foot X-rays were obtained to measure the preoperative parameters, including the standing talo-first metatarsal angle and talonavicular coverage angle in the AP view and talo-first metatarsal angle and calcaneal pitch angle in the lateral view.

In bilateral cases, we operated each side separately with a 4 to 6-month interval.

### Surgical technique

Surgeries were done with patients in the supine position under general anesthesia. Antibiotic prophylaxis, third generation cephalosporins, was used two hours before the time of anesthesia induction at a dose of 50 mg/kg. A pneumatic tourniquet was used in all patients.

### Calcaneal lengthening osteotomy

**Fig. 1:**
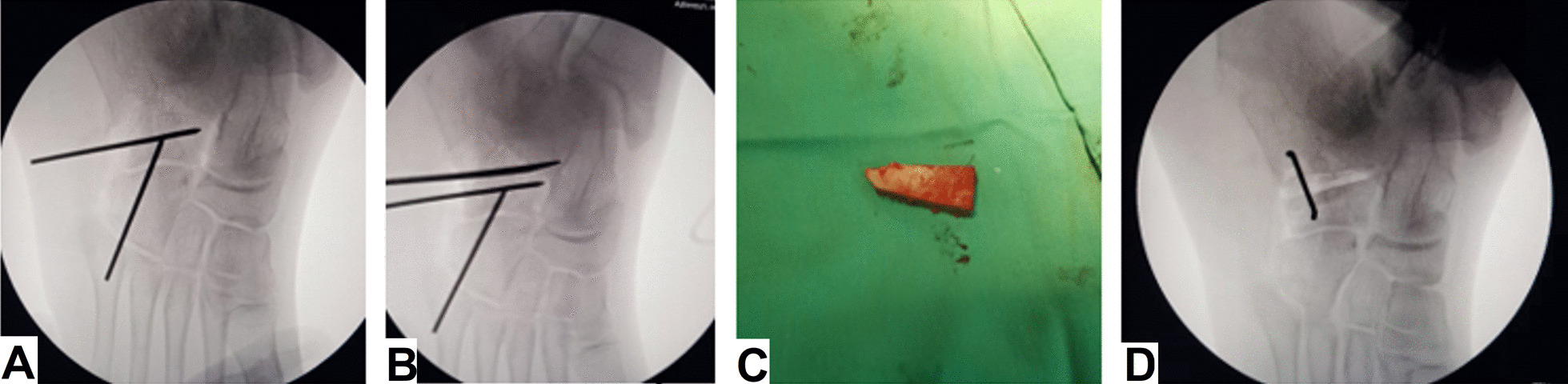
Surgical steps on the lateral side. **A** Stabilization of the calcaneocuboid joint and insertion of a reference wire for the osteotomy site. **B** Osteotomy of the calcaneus using the oscillating saw. **C** A harvested trapezoidal tricortical iliac graft. **D** Application of the graft and stabilization with a stable

A 5 to 6 cm lateral longitudinal incision was centered over the calcaneocuboid joint. The sural and superficial branches of the peroneal nerve were protected. Dissection took place between the extensor digitorum brevis dorsally and the peroneal muscles plantarly. The calcaneocuboid joint (CCJ) was determined without joint capsule opening. The CCJ was fixed using 1.5 mm K-wire inserted from distal to proximal and stopped before the planned osteotomy site to prevent subluxation of the CCJ while opening the osteotomy site. Z-lengthening of the peroneus brevis was performed. Localization of the osteotomy site was done by the insertion of a reference K-wire about 1–1.5 cm proximal to the calcaneocuboid joint and parallel to it. The osteotomy was done between the anterior and middle facet of the calcaneus using an oscillating saw just proximal to the reference wire and under C-arm control. The osteotomy site was then opened using a lamina spreader up to the maximum opening to determine the width of the graft needed. A tricortical trapezoidal-shaped bone graft was taken from the ipsilateral iliac crest. The width of the graft ranged from 8 to 12 mm at one edge and 4 to 6 mm on the opposite edge. The graft was inserted and well-fitted into the osteotomy site. Although the graft was inherently stable, additional stability was added using a single staple to prevent dislodgement of the graft, Fig. 1.

### Tibialis anterior rerouting

**Fig. 2:**
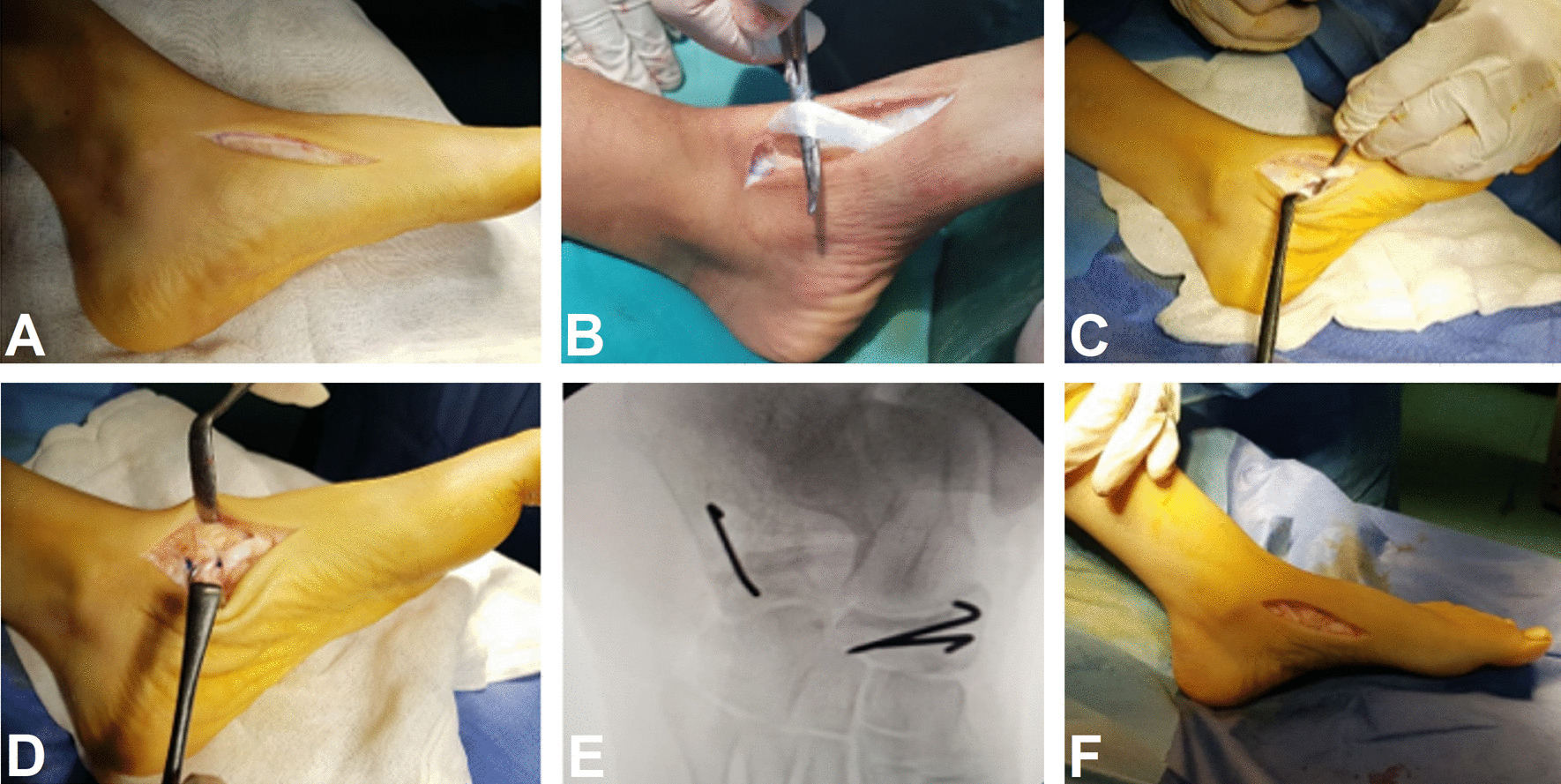
Tibialis anterior rerouting. **A** Medial skin incision. **B** Releasing the tendon. **C** Pulling the tendon to the planter surface of the navicular. **D** Keeping the tendon in place with sutures. **E** K-wires insertion into the navicular to keep the tendon in place. **F** Final corrected foot shape

An 8 to 10 cm incision was made just medial to the tibialis anterior tendon from the first metatarsal base distally to the talonavicular joint proximally. The incision was deepened, and the tibialis anterior tendon was freed up along its route, keeping it attached to its insertion. A U-shaped osteoperiosteal flap was made on the medial side of foot, extending over the capsule and overlying the talonavicular joint, navicular bone, navicular-medial cuneiform joint and medial cuneiform bone keeping the flab attached at the planter side. A gutter was then created beneath the navicular tuberosity, and the tibialis anterior tendon was then pulled down into this gutter and stitched to soft tissues beneath the talar head. Two K-wires were inserted into the navicular as anchors to prevent slippage of the tendon back till soft tissue healing. The U-shaped osteoperiosteal flap was then sutured back into its place over the tibialis anterior tendon keeping the tendon in the gutter and preventing it from dislocating, Fig. 2.

### Fractional lengthening of the gastrocnemius muscle

**Fig. 3:**
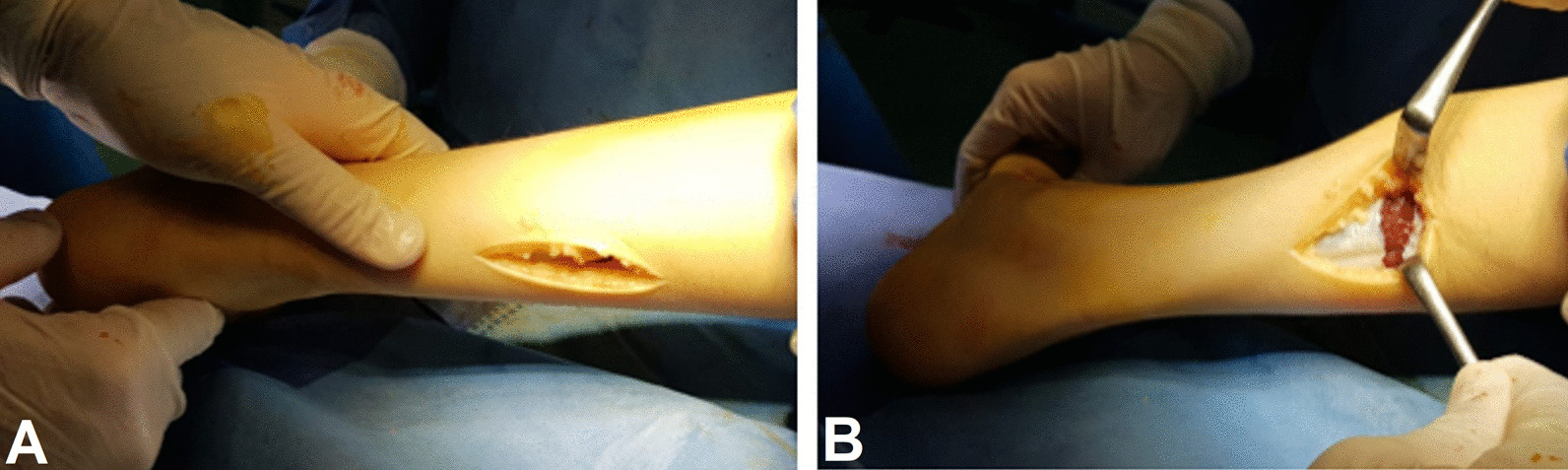
Fractional lengthening of the gastrocnemius muscle. **A** Posterior skin incision. **B** Fractional lengthening of the tendinous part of the gastrocnemius muscle at the musculotendinous junction

After completion of calcaneal lengthening and tibialis anterior rerouting, testing for ankle dorsiflexion was done. The ankle should be dorsiflexed to 10–15 degrees. In cases with ankle contracture, the Silfverskiold test was used to evaluate the cause of the contracture, whether it was gastrocnemius muscle or the Achilles tendon. Patients with positive Silfverskiold test underwent fractional lengthening of the gastrocnemius muscle through a separate posterior approach, Fig. 3.

After completion of the surgical procedures, the tourniquet was deflated, and proper hemostasis was done before wound closure and application of a below well-padded knee cast.

### Postoperative care

Immediate postoperative foot X-rays were done to assess the degree of correction of the radiological parameters. Postoperative antibiotic prophylaxis was given for 3 days in the form of third generation cephalosporins with a dose of 50 mg/kg/day. Patients were discharged from the hospital on the second day postoperative. Follow-up was done in the outpatient clinic two weeks postoperatively for wound assessment, stitches removal, and application of a new below-knee cast. Six weeks later, the cast was removed, and new foot X-rays were done to assess the healing of the calcaneal osteotomy. Below-knee walking cast was then applied and partial weight bearing was allowed for 4 weeks. This was followed by cast removal and full weight-bearing. Regular follow-up visits were advised every 6 months.

The American Orthopaedic Foot and Ankle Society (AOFAS) score [22] was utilized to assess the functional results of patients. Results were excellent with a score of 85 or more, good with a score of 75–84, fair when the score was 60–74, and bad when the score was less than 60.

### Statistical analysis

Data were analyzed using IBM SPSS software package version 20.0. **(**Armonk, NY: IBM Corp**)**. Continuous variables were represented as mean ± SD. Qualitative data were represented as numbers and percentages. Wilcoxon signed-rank test was utilized to compare quantitative variables which were not normally distributed. Results were judged significant at a 5% level.

## Results

### Demographics and baseline characteristics

This study included 28 feet in 16 patients, with a mean age of 11.6 ± 2.1 (range, 9–14.5) years. Eight (50%) patients were males and eight (50%) were females. The affected side was bilateral in 12 (75%) patients, right in 3 (18.8%) patients, and left in 1 (6.2%) patient. The main complaint was pain in 13 (46.4%) feet, cosmetic issues in 3 (10.7%) feet, and pain and cosmetic issues in 12 (42.9%) feet.

The average preoperative AOFAS score was 51.6 ± 5.5 (range, 42–61).

In 25 (89.3%) feet, there was ankle contracture with a positive Silfverskiold test that necessitated fractional lengthening of the gastrocnemius muscle through a separate posterior approach. The remaining 3 (10.7%) feet had no ankle contracture, Table 1.

**Table 1:** Demographics and baseline characteristics of the included patients

Characteristics	Value (*n* = 16 patients, 28 feet)
Age, years (mean ± SD)	11.6 ± 2.1
Gender (*n*, %)	
Male	8 (50%)
Female	8 (50%)
Affected side (*n*, %)	
Bilateral	12 (75%)
Right	3 (18.8%)
Left	1 (6.2%)
Complaint (*n*, %)	
Pain	13 (46.4%)
Cosmetic issue	3 (10.7%)
Pain and cosmetic issues	12 (42.9%)
Preoperative AOFAS score (mean ± SD)	51.6 ± 5.5

*AOFAS*—American Orthopaedic Foot and Ankle Society

### Functional and radiological outcomes

The average follow-up duration was 38.9 ± 10 (range, 24–60) months. The average AOFAS score increased from 51.6 ± 5.5 (range, 42–61) preoperatively to 85.3 ± 10.2 (range, 56–96) at final follow-up, *P* < 0.001. Excellent results were obtained in 17 (60.7%) feet, good results in 8 (28.6%) feet, fair results in 1 (3.6%), and poor results in 2 (7.1%) feet. The 3 cases with fair and poor results complained of some degrees of pain, limited recreational activities, and hindfoot motion restriction. Achilles tendon lengthening was not performed in those 3 feet.

**Fig. 4:**
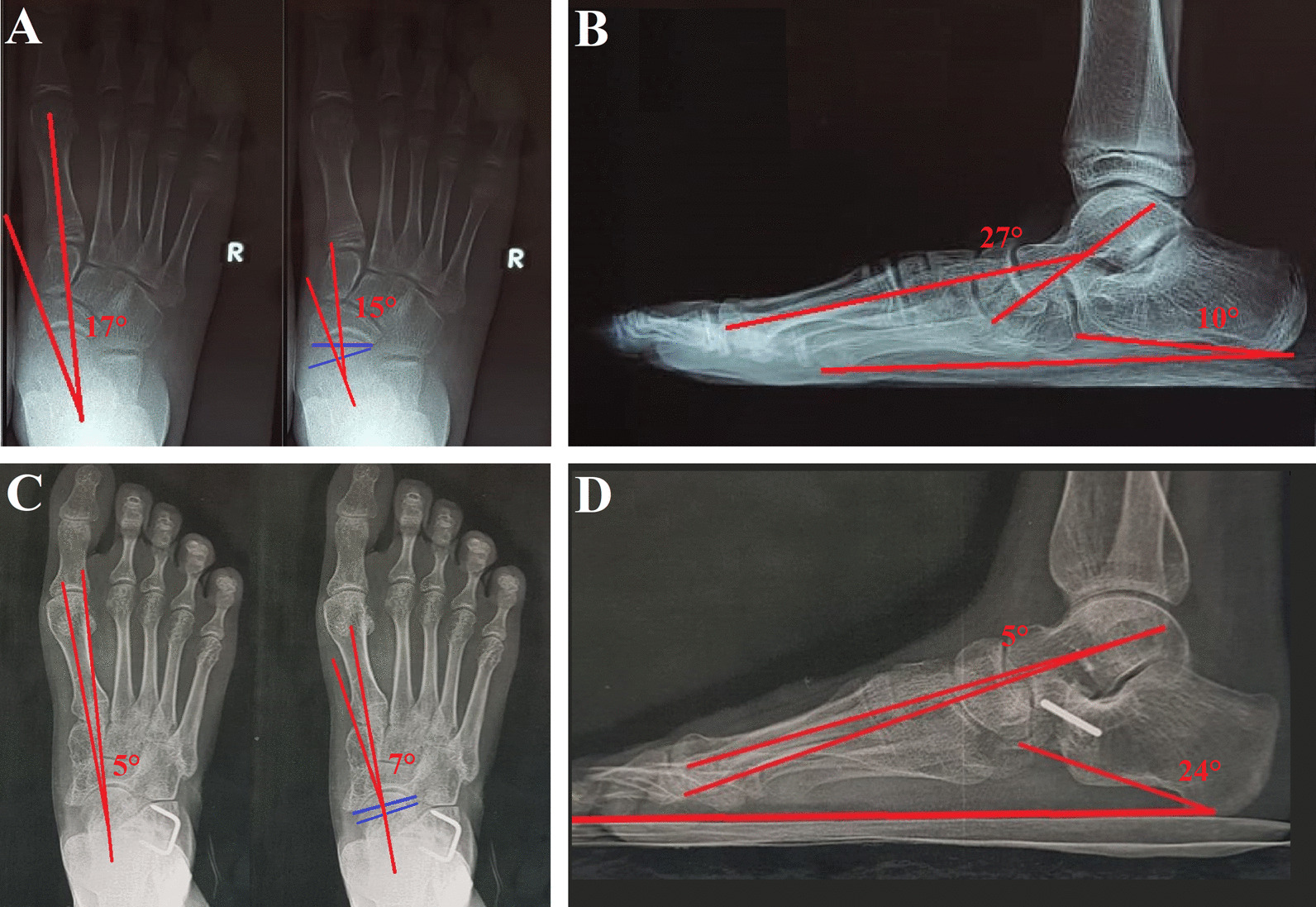
AP and lateral X-ray images at the weight-bearing position of a 12-year-old female child. **A** Preoperative AP talo-first metatarsal angle (17°) and talonavicular coverage angle (15°). **B** Preoperative lateral talo-first metatarsal angle (27°) and calcaneal pitch angle (10°). **C** Postoperative AP talo-first metatarsal angle (5°) and talonavicular coverage angle (7°). **D** Postoperative lateral talo-first metatarsal angle (5°) and calcaneal pitch angle (24°)

The talonavicular coverage angle decreased from 13.6 ± 4.4° (range, 5–25) preoperatively to 3.9 ± 3° (range, 0–10) postoperatively, *P* < 0.001. The AP talo-first metatarsal angle reduced from 16.9 ± 4.4° (range, 10–25) preoperatively to 4.5 ± 3.6° (range, 0–15) postoperatively, *P* < 0.001. The lateral talo-first metatarsal angle decreased from 19.2 ± 4.9° (range, 10–30) preoperatively to 4.6 ± 3.2° (range, 0–12) postoperatively, *P* < 0.001. The calcaneal pitch angle increased from 9.6 ± 1.9° (range, 5–13) preoperatively to 23.8 ± 4.8° (range, 12–30) postoperatively, *P* < 0.001, Table 2, Fig. 4.

**Table 2:** Preoperative and postoperative radiological parameters

Radiological parameters	Preoperative	Postoperative	*P* value
Talonavicular coverage angle (normal < 7°)			
Mean ± SD	13.6 ± 4.4°	3.9 ± 3°	< 0.001
Range	(5°–25°)	(0°–10°)	
AP talo-first metatarsal angle (normal 7° ± 4°)			
Mean ± SD	16.9 ± 4.4°	4.5 ± 3.6°	< 0.001
Range	(10°–25°)	(0°–15°)	
Lateral talo-first metatarsal angle (normal 0°–4°)			
Mean ± SD	19.2 ± 4.9°	4.6 ± 3.2°	< 0.001
Range	(10°–30°)	(0°–12°)	
Calcaneal pitch angle (normal 10°–25°)			
Mean ± SD	9.6 ± 1.9°	23.8 ± 4.8°	< 0.001
Range	(5°–13°)	(12°–30°)

### Complications

No major complications developed. Three feet got superficial wound infection which was managed by dressing and antibiotics according to culture and sensitivity. Five feet developed hardware irritation from the medial K-wires that were removed after a sufficient period for complete soft tissue healing (4–6 months postoperatively).

## Discussion

Flexible flatfoot is a frequent three-dimensional deformity in children and adolescents which can be asymptomatic or present with pain and gait disturbances [23]. The treatment aims to relieve the symptoms and to realign the foot [24]. Corrective surgery is sometimes necessary for moderate or severe deformities with failed conservative measures [10, 20, 25]. Joint arthrodesis is not advised for correction of flexible flatfoot as it can lead to stress shifting to adjacent joints and early arthritic changes [1].

As a result of their unreliable results and deformity recurrence, isolated soft tissue procedures are no longer advocated [8]. Combined bony and soft tissue procedures are most effective in correcting the deformity in all three planes [1, 20, 21, 24, 26]. By correcting the deformity, the foot kinematics improve during weight-bearing, which reduces the rapid progression of the deformity, which could cause degenerative changes to the foot and the need for future arthrodesis [16, 27].

Evans lateral calcaneal lengthening osteotomy can address the collapsed medial arch and heel eversion, but this osteotomy alone ignores other elements of the flatfoot deformity [28, 29]. Several medial column soft tissue procedures have been reported, such as tibialis anterior transfer to the navicular and tibialis anterior or posterior tenodesis [30].

In our study, a reconstructive technique of tibialis anterior rerouting combined with calcaneal lengthening osteotomy was used. A trapezoidal iliac graft was used in the calcaneal osteotomy to keep the lengthening, as advised by Mosca [15]. Despite the graft being inherently stable after application, additional fixation with a stable was done to save guard against dislodgement of the graft, which had not occurred in any case.

Kissel and Blacklidge [31] transferred the tibialis anterior tendon via the talus from lateral dorsal to medial plantar, with suturing of the remaining tendon to the soft tissues.

In our study, the distal 10–15 cm of the tibialis anterior tendon was mobilized and redirected in an L course without disruption of the tendon insertion. The horizontal limb acted as a strong plantar ligament, and the vertical limb was stitched to the undersurface of talonavicular joint to support the subluxated joint. After its rerouting, the tibialis anterior can still act as an ankle dorsiflexor, as it mainly functions during the swing phase [32].

In this study, Achilles tendon lengthening was done in 25 feet through fractional lengthening of the musculotendinous junction. Achilles tendon contracture often coexists with symptomatic flatfoot, preventing normal ankle dorsiflexion during midstance [33]. In these cases, Achilles tendon lengthening is necessary to allow the ankle to dorsiflex at least 10–15 degrees [34, 35]. In our study, Achilles tendon lengthening was not performed in 3 cases that had fair and poor results. Therefore, we advise that ankle equinus should be thoroughly examined after correction of the deformity for lengthening Achilles tendon or Gastrocnemius fractional lengthening in positive Silfverskiold test.

In our study, the average AOFAS score increased significantly from 51.6 ± 5.5 preoperatively to 85.3 ± 10.2 at the last follow-up. Our results were comparable to previous studies that reported other reconstructive methods. Oh et al. [36] reported the outcomes of combined reconstruction of the symptomatic flexible flatfoot in adolescents using medializing calcaneal osteotomy and lateral column lengthening. The average AOFAS score increased significantly from 49.1 preoperatively to 93.4 at last follow-up [36]. Viegas et al. [30] reported significant improvement of the average AOFAS score from 68.59 preoperatively to 96.35, following Evans calcaneal osteotomy combined with medial split tibialis anterior tendon transfer, in 34 flexible planovalgus feet, with an average follow-up of 24.9 months.

In our study, all the radiological parameters improved significantly postoperatively, including the AP and lateral talo-first metatarsal angle, talonavicular coverage angle and calcaneal pitch angle. Similarly, Oh et al. [36] and Viegas et al. [30] reported improvement in those radiological procedures following their technique.

Lateral calcaneal lengthening osteotomy must be performed with caution. Li et al. [37] conducted a cadaveric study on the optimal size of graft size and reported that a 6 mm graft was not associated with any significant calcaneal shift, while grafts of 8 mm or more led to significant anterior and posterior shift of two calcaneal segments and posterior facet impingement.

The idea of tibialis anterior rerouting resembles Young’s tenosuspension procedure [38], which reroutes the tibialis anterior tendon through a navicular key-hole slot. In our study, tibialis anterior rerouting was performed without separating the tendon from its insertion. The tibialis anterior tendon was not rerouted into the talar head, but it was rerouted plantarly beneath the navicular bone to support the talonavicular joint, acting as a second spring ligament to support the medial arch. As the distal part of the tibialis anterior tendon was left attached to its insertion, rerouting of the tendon plantarly increased its tension and thus creating a supination moment of the foot without affection of its function as a dorsiflexor of the tibiotalar joint during the swing phase and act as a static stabilizer of the talonavicular joint preventing the dropping of the medial arch during the stance phase.

The rerouting of the tibialis anterior tendon beneath the navicular, however, may increase forefoot supination postoperatively. This soft tissue procedure weakens the dorsiflexion power of the tibialis anterior, leading to compensatory overactivity of toe extensors and a mechanical advantage to the plantar flexion power of the peroneus longus that might evolve over time [39].

The tibialis anterior tendon mainly functions during the swing phase, not the stance phase [32]. Therefore, it is questionable whether this procedure has an active effect on the rise of the medial arch, which is more likely done by the unantagonized long peroneal function. To investigate the possible long-term drawbacks and biomechanical changes of this procedure, a long-term follow-up study is needed.

The procedure presented in this study was adopted from El-Tayeby study [21], that reported treatment of severe flexible flatfoot with combined Evans calcaneal osteotomy and tibialis anterior rerouting in addition to navicular-first cuneiform wedge resection and arthrodesis for shortening of the medial column of the foot. We did not perform naviculocuneiform arthrodesis as this additional invasive bony procedure may result in limitation of foot motions, overcorrection of the deformity, and unexpected long-term outcomes.

This study has some limitations, such as the relatively low number of operated feet and the relatively non-adequate follow-up period that is not enough to address the long-term outcomes of this procedure on foot, such as degenerative arthrosis. We did not perform footprint analysis by a pedograph as it is costly and not widely available in our country enough to be performed on a regular basis. The absence of comparison between the results of this procedure and other procedures is another limitation of the study. However, the combined technique described in this study is a good option for addressing and maintaining all components of flatfoot deformity.

## Conclusion

Surgical reconstruction of symptomatic flexible flatfoot with lateral column lengthening and tibialis anterior rerouting has good radiological and clinical outcomes with a considerably low complication rate.

## Data Availability

The dataset analyzed in this study is available from the corresponding author on request.

## References

[1] Chien BY, Vosseller JT, Younger A, Greisberg J (2023). Surgical treatment of the flexible, progressive collapsing foot: deformities, definitions, and decisions. Instr Course Lect.

[2] Lamm BM, Knight J, Ernst JJ (2022). Evans calcaneal osteotomy: assessment of multiplanar correction. J Foot Ankle Surg.

[3] Kim JY, Kim SA, Kim Y, Hwang I, Heo NH (2023). Radiologic changes of long term foot insole use in symptomatic pediatric flatfoot. Medicine.

[4] Bohm H, Dussa CU (2023). Clinical, radiographic and gait parameters associated with medial arch pain in the flexible pediatric flatfoot. J Foot Ankle Surg.

[5] Alsiddiky AM, Alsubaie AA, Almuhanna AO, Alsubaie NM, Alsaleh FA, Alhazzani HM (2023). Satisfactory outcomes of post-operative subtalar extra-articular arthroereisis in juvenile flexible flat foot. Saudi Med J.

[6] Ghaznavi A, Mahdavi SM, Moghtadaei M, Taheri SN, Yeganeh A, Karimpour A (2022). Calcaneostop provides favorable short-term outcomes in patients with flexible flatfoot. Med J Islam Repub Iran.

[7] Smith C, Zaidi R, Bhamra J, Bridgens A, Wek C, Kokkinakis M (2021). Subtalar arthroereisis for the treatment of the symptomatic paediatric flexible pes planus: a systematic review. EFORT Open Rev.

[8] Mosca VS (2010). Flexible flatfoot in children and adolescents. J Child Orthop.

[9] Payehdar S, Taheri A, Tahririan M (2023). Impact of night orthotic managements on gastroc-soleus complex tightness in pediatric with flexible flatfoot: systematic review. Prosthet Orthot Int.

[10] Bouchard M, Mosca VS (2014). Flatfoot deformity in children and adolescents: surgical indications and management. J Am Acad Orthop Surg.

[11] Hosny GA, Hussein MA, Zaghloul KM, El-Mowafi H, Khalifa AA (2023). Lateral column lengthening (LCL) using a rectangular shape graft for managing symptomatic flexible flatfoot showed acceptable early functional and radiological outcomes. Foot.

[12] Jerosch J, Schunck J, Abdel-Aziz H (2009). The stop screw technique–a simple and reliable method in treating flexible flatfoot in children. Foot Ankle Surg.

[13] Madden CM, Mahan KT (2023). An update on pediatric flatfoot. Clin Podiatr Med Surg.

[14] Evans D (1975). Calcaneo-valgus deformity. J Bone Joint Surg Br.

[15] Mosca VS (1995). Calcaneal lengthening for valgus deformity of the hindfoot. Results in children who had severe, symptomatic flatfoot and skewfoot. J Bone Joint Surg Am..

[16] Kumar S, Sonanis SV (2017). Lateral column lengthening for adolescent idiopathic pes planovalgus deformity—systematic review. J Orthop.

[17] Lima TC, Volpon JB (2018). Calcaneal lateral column lengthening osteotomy for symptomatic flexible flatfoot in children and adolescents: a systematic review. Rev Col Bras Cir.

[18] Ford SE, Scannell BP (2017). Pediatric flatfoot: pearls and pitfalls. Foot Ankle Clin.

[19] Samaila E, Bonetti I, Bruno C, Argentini E, Magnan B (2016). Navicular tenosuspension with anterior tibialis tendon (Young procedure) associated to calcaneo-stop for the treatment of paediatric flexible flatfoot: clinical and ultrasound study. Acta Biomed.

[20] Bouchard M, Ross TD (2021). Bony procedures for correction of the flexible pediatric flatfoot deformity. Foot Ankle Clin.

[21] el-Tayeby HM (1999). The severe flexible flatfoot: a combined reconstructive procedure with rerouting of the tibialis anterior tendon. J Foot Ankle Surg.

[22] Kitaoka HB, Alexander IJ, Adelaar RS, Nunley JA, Myerson MS, Sanders M (1994). Clinical rating systems for the ankle-hindfoot, midfoot, hallux, and lesser toes. Foot Ankle Int.

[23] Pfeiffer M, Kotz R, Ledl T, Hauser G, Sluga M (2006). Prevalence of flat foot in preschool-aged children. Pediatrics.

[24] Blitz NM, Stabile RJ, Giorgini RJ, DiDomenico LA (2010). Flexible pediatric and adolescent pes planovalgus: conservative and surgical treatment options. Clin Podiatr Med Surg.

[25] Sheikh Taha AM, Feldman DS (2015). Painful flexible flatfoot. Foot Ankle Clin.

[26] DeVries JG, DeCarbo WT, Scott RT, Bussewitz B, Thompson M, Hyer CF (2022). Soft tissue reconstruction and osteotomies for pes planovalgus correction. Clin Podiatr Med Surg.

[27] Roche AJ, Calder JD (2012). Lateral column lengthening osteotomies. Foot Ankle Clin.

[28] De Luna V, De Maio F, Caterini A, Marsiolo M, Petrungaro L, Ippolito E (2021). Surgical treatment of severe idiopathic flexible flatfoot by evans-mosca technique in adolescent patients: a long-term follow-up study. Adv Orthop.

[29] DeYoe BE, Wood J (2005). The Evans calcaneal osteotomy. Clin Podiatr Med Surg..

[30] Viegas GV (2003). Reconstruction of the pediatric flexible planovalgus foot by using an Evans calcaneal osteotomy and augmentative medial split tibialis anterior tendon transfer. J Foot Ankle Surg.

[31] Kissel CG, Blacklidge DK (1995). Tibialis anterior transfer "into talus" for control of the severe planus pediatric foot: a preliminary report. J Foot Ankle Surg.

[32] Knutsen AR, Avoian T, Sangiorgio SN, Borkowski SL, Ebramzadeh E, Zionts LE (2015). How do different anterior tibial tendon transfer techniques influence forefoot and hindfoot motion?. Clin Orthop Relat Res.

[33] Ueki Y, Sakuma E, Wada I (2019). Pathology and management of flexible flat foot in children. J Orthop Sci.

[34] Costa ML, Logan K, Heylings D, Donell ST, Tucker K (2006). The effect of achilles tendon lengthening on ankle dorsiflexion: a cadaver study. Foot Ankle Int.

[35] Carr JB, Yang S, Lather LA (2016). Pediatric pes planus: a state-of-the-art review. Pediatrics.

[36] Oh I, Williams BR, Ellis SJ, Kwon DJ, Deland JT (2011). Reconstruction of the symptomatic idiopathic flatfoot in adolescents and young adults. Foot Ankle Int.

[37] Li S, Myerson M, Travascio F, Albarghouthi A, Bang K, Latta L (2019). Investigation of lateral column lengthening osteotomy on the movement of the calcaneus: a cadaveric study. Foot Ankle Orthop.

[38] Young C, Charles S (1939). Operative treatment of pes planus. Surg Gynecol Obstet.

[39] Jacobs AM (2007). Soft tissue procedures for the stabilization of medial arch pathology in the management of flexible flatfoot deformity. Clin Podiatr Med Surg.

